# Infant Feeding1Read before the Buffalo Academy of Medicine, October 28, 1902.

**Published:** 1902-12

**Authors:** A. J. Colton

**Affiliations:** Pediatrist to Buffalo Hospital Sisters of Charity, 41 Otis Place.


### Infant Feeding.[1]

A. J. COLTON, M.D.,

Pediatrist to Buffalo Hospital Sisters of Charity.

BY FAR the most important subject in pediatrics today, is that relating to the feeding of infants. Without proper nourishment, not only is the infantile mortality greatly increased, but should it escape the ravages of death its whole life, character

1. Read before the Buffalo Academy of Medicine, October 28, 1902.

and physical being may be entirely changed. The importance of infant-feeding is exemplified by the great number of baby foods on the market—all lauded by the manufacturers to be equal to or even better than the breast milk, but none proving by the test of experience to be in any way ideal or meet the requirements of the nursing babe. It is true that some babes will exist on these foods and a few apparently thrive; but a far greater percentage will develop marasmus, rachitis, scurvy and improper development of teeth and bone; besides the various gastrointestinal disturbances.

The number and variety of baby foods on the market, are like the remedies recommended in diseases for which there is no specific—innumerable—a most excellent proof that none are ideal or meet the requirements in a majority of cases.

As an infant is fed usually in one of three ways, I have made three classifications of infant feeding, viz: (1) from the maternal breast; (2) by a wet nurse; (3) from the bottle, or what is sometimes called artificial or hand-feeding. A fourth might be mentioned, called mixed feeding—partly from the breast and bottle. Other methods are and have been used.

Ancient history tells us that Romulus and Remus were nursed from the breast of a she wolf. It is also reported in medical literature, that the male breasts have been known to develop sufficiently to secrete enough milk for the nourishment of the infant. Of these unusual varieties of feeding, I shall not discuss.

The best and most natural method of feeding infants is from the maternal breast; but this unfortunately cannot always be resorted to. The social conditions of the mother may be such that she cannot or will not nurse her babe. The mother who depends on her own livelihood for the support of herself and family, would find it out of the question to nurse her babe; and the society woman finds it also, in her way of thinking, out of the question to nurse her new-born. Or, again, the quantity of the mother's milk may be so deficient that she is compelled to resort to other modes of feeding; again, the quality of the milk—which is rare—may be such that the baby is in constant torment from the cramps or colic produced by it. In such cases, other modes of feeding should be resorted to.

In maternal feeding, the points of importance are, when the milk is of the proper quality and quantity, the length of the interval between feedings, and the length of time the baby should be permitted to nurse at the breast. No iron clad rules can be laid down for all cases; but, as a general rule, the babe should be put to the breast as soon as the mother is over the fatigue of labor—four to eight hours after birth. For the first three days, or until the breasts fill, the interval should be every four hours, alternately with each breast. It would be worse than useless to nurse oftener at this time, as the breasts contain no milk, and but a small quantity of colostrum, and more frequent nursing at the start is apt to cause sore nipples and dire consequences may result therefrom. After three or four days, when the milk is secreted in sufficient quantity, it may be put to the breasts every two hours, from 7 a.m. to 10 p.m., with two nursings through the night. This is to be continued until the baby is six weeks old, when feedings should be lengthened to every two and one-half hours, with one night feeding, until the fourth month; then every three hours, with one night feeding, and after the fifth month, no night nursing whatsoever. It is important that the babe be awakened at its regular feeding time during the day to nurse, that it may form correct habits and thus prevent indigestion from over distention of the stomach. The nipples should be washed with a saturated solution of borax water or boracic acid solution, before and after nursing, thus preventing, to a large extent, both sore nipples and the common forms of stomatitis.

No. 2, or wet-nursing, I shall dispose of in a few words. If the wet nurse be physically, mentally and morally qualified and her milk is of the proper quantity and quality, and expense no consideration, then, of course, a wet nurse should be preferred next to the maternal breast; but these qualities are rarely attained in a wet nurse. If her physical condition be correct and she is free from syphilis, tuberculosis or any other transmittible or infectious disease, her morals may be such that it would be unwise for her to have the care of a young infant, which she would necessarily be obliged to have more or less. Masturbation is frequently taught the infant by the wet nurse, either by her depraved nature or from her desire to appease a crying infant. Her physical and moral conditions may be all right and her personal appearance or conditions such as filthiness in habits, slovenliness or ugly features should disqualify her. All these may be correct and the secretion of her milk will be made variable in quantity and quality in many cases, from her remorse over the conditions which caused her to become a wet nurse, or the worry caused by her own infant being deprived of its mother's milk. These factors all cause a disturbance, both in the quantity and quality of the milk and affect the digestion of the infant in a like degree. There are scores of other things not here mentioned, to disqualify a wet nurse. Mixed feeding may be resorted to when the mother's milk is deficient in quantity or to relieve the mother of night nursing, and with the most happy and beneficial results, both to the mother and infant.

The method of feeding which is of most importance to the infant deprived of breast milk and the one which requires of us, as physicians, the greatest amount of study, care and skill, is artificial or bottle-feeding, sometimes called hand-feeding. In No. 3, or artificial feeding, there are several fundamental principles which must be constantly borne in mind: (1) the food must contain the same constituents as found in woman's milk, viz.: fats, sugars, proteids and inorganic salts; (2) these constituents must be present in about the same proportion as found in good healthy woman's milk; (3) as nearly as possible, the different constituents should resemble woman's milk, both as to chemical composition and their behavior to the digestive fluids.

How decided is the difference in the action of pepsin in the proteids of cow's milk and that of human milk; in the one, the curd is a thick, rubbery, tenacious substance; in the other, a light, thin, flocculent curd. The properties of human milk are not found in any of the shop foods. Human milk is composed of 13 parts solids and 87 parts water. The solids are the fats, sugars, proteids and inorganic salts. The sugar or carbohydrates is lactose or sugar of milk. The proteids or albumenoids are the curd. The cream is the fat or the hydrocarbons. These elements are all essential for the growth of the various organs, bones, nerves, fat of the body and for the production of heat. The sugar is needed for the production of heat and making fat; proteids are needed for the growth of the cells of the body, such as those of the blood and various organs and muscles. The fats are needed for the growth of bone, nerves, fat of body and production of heat. The salts are required for bone construction.

There are many substitutes for human milk sold in the stores, but the only substitute which I shall consider, is a properly modified cow's milk. I mean by the modification of cow's milk, the changing of the proportions of the different ingredients until they resemble human milk, the important changes required being to diminish the proteids and increase the sugar. The essentials of a cow's milk used in infant-feeding are, that it shall be fresh, clean and from healthy cows. It is better to have the milk from a herd than from a single cow, as the milk from a single cow varies from day to day, while that from a herd remains practically the same. There are now in most every large city of the United States, laboratories for the modification of cow's milk on physicians' prescriptions. On account of the expense, people with moderate means are unable to use it. For this class of people, I recommend the home modification of cow's milk, and with this method I have fully as good results when the modification is properly carried out. It can be done by anyone with ordinary intelligence, when properly instructed. The milk of the cow, ewe, goat, mare and ass, contain the same ingredients practically, as found in human milk, but differ as to the proportion of their constituents. The milk of the mare and ass most nearly correspond to human milk of any of the mammals, but on account of the difficulty in obtaining it, it is rarely used. Cow's milk being the most available, is the one used most commonly as a substitute for human milk.

The principal reason for modifying cow's milk are because it contains about three times as much proteids or curd as human milk, and only about one-half the amount of sugar; besides the digestive fluids of the infant produce vastly different effects on the proteids of cow's and human milk. The one is made into a tough and tenacious curd, while the other is made into a light and flocculent curd. Furthermore, the reaction of human milk is always alkaline, while that of cow's milk is persistently acid.

The following comparative table of human and cow's milk, shows the reasons for modification.

| | HUMAN MILK. | COW'S MILK. |
|---|---|---|
| Reaction | Alkaline | Acid |
| Specific Gravity | 1,031 | 1,029 |
| Fats | 4 per cent. | 4 per cent. |
| Sugars | 7 per cent. | 4.3 per cent. |
| Proteids | 1½ per cent. | 4 per cent. |
| Salts | 0.2 per cent. | 0.7 per cent. |
| Water | 87 30 per cent. | 87 per cent. |
| Bacteria | Usually none | Always more or less persistent. |

The fats are the only ingredients which are alike. The proteids must be greatly reduced, the sugars increased, the salts reduced, the acidity overcome, and as far as possible, the bacteria destroyed. The proteids may be diminished by diluting the milk according to the age of the child. To give the proper proportion of proteids, cow's milk should be diluted for an infant under two weeks, five times; from 2 to 6 weeks, four times; from 6 to 12 weeks, three times; from 12 to 16 weeks, one and one-half times; from 16 to 40 weeks, once; and a baby one year old, usually not at all.

I have devised a table, modified from Holt, of five formulæ, showing the ingredients, administration and preparation of modified milk for infant feeding, from birth to 10 months, as follows:

| | FORMULÆ. | I. | II. | III. | IV. | V. |
|---|---|---|---|---|---|---|
| | Age | 3 to 14 days. | 2 to 6 weeks. | 6 to 12 weeks. | 12 to 20 weeks. | 5 to 10 months |
| Ingredients. | Milk | 1½ oz. | 3 oz. | 4 oz. | 10 oz. | 15¾ oz. |
| | Cream | 1½ oz. | 3 oz. | 4 oz. | 4 oz. | 5¼ oz. |
| | Milk-sugar | 1¼ oz. | 1¾ oz. | 2 oz. | 2¼ oz. | 2¾ oz. |
| | Lime water | 1½ oz. | 1½ oz. | 1½ oz. | 1½ oz. | 2 oz. |
| | Water | 15½ oz. | 20½ oz. | 22½ oz. | 19½ oz. | 19 oz. |
| Preparation and Administration. | Amount at each feeding | 1-2½ oz. | 2-3¼ oz. | 3-4½ oz. | 4-6 oz. | 5-8 oz. |
| | Interval between feedings | 2 hours. | 2 hours. | 2½ hrs. | 3 hours. | 3 hours. |
| | Number of feedings in 24 hours | 10 | 9 | 8 | 7 | 6 |
| | Number of night feedings from 10 p.m. to 7 a.m. | 2 | 2 | 1 | 1 | 0 |
| | Amount for 24 hours | 10-25 oz | 20-32 oz. | 24-36 oz. | 28-42 oz. | 30-48 oz. |
| | Number of dilutions of milk top | 5 times. 12 % | 4 times. 12 % | 3 times 12 % | 1½ times 9 % | once 8% |

NOTE—Twelve per cent. top milk is the upper 1-5 of milk, which has stood in a cool place for 5 hours; 9 per cent., the upper 1-4; 8 per cent., the upper 1-3. Top milk is equal to the milk and cream combined, in the above formulæ.

PREPARATION—Syphon the milk from the bottom of the bottle after it has stood on ice, or in a cool place, for from 4 to 5 hours, until 1-5 remains; take of this the amount required for 24 hours and add to the sugar of milk solution, which is made by adding the required amount of sugar of milk to boiling water. Add the lime water last. Next pour the amount of one feeding into each of the feeding bottles required for 24 hours; put stoppers in loosely and pasteurise for half hour. After pasteurising, push stoppers in well and cool by placing bottles, first, in tepid water, then, in very cold water, after which put in a cool place.

On account of the greater indigestible qualities of the curd, in cow's milk, it must be reduced far below that of human milk, as the table will show. Instead of reducing the proteids to 1½ per cent., the percentage in human milk, for an infant from 2 to 14 days, it must be reduced to 6-10 per cent. and in some cases still more, and as the infant becomes accustomed to it, it can be gradually increased. Not until the babe is 12 weeks old, is it able to digest the curd in cow's milk, at the same percentage found in human milk, 1½ per cent., the curd of cow's milk being so much more difficult to digest. In some instances, I have been obliged to remove it altogether and make a mixture of whey and cream. The curd is easily separated from the milk by the use of pepsin (Fairchild's), about one dram to a pint. The whey and cream are then used for feeding. The quantity of sugar of milk used in whey and cream is only about one-half as much, because in using the whey, the greater percentage of sugars is retained and only the difference between that of cow's and human milk is to be added. In order then to modify cow's milk, we must have milk, cream, sugar of milk, lime water and water. It is essential that the cream and milk be fresh and not older than from 12 to 18 hours. Unless you are able to obtain centrifuged cream, it is better to use what is termed by Holt, "top milk." Instead of using 1½ ounces each of cream and milk, as in formula No. 1, you may use 3 ounces of 12 per cent. top milk. The upper 1-5 of the bottle of milk, standing in a cool place for five hours, will contain 12 per cent. butter fat and 3 per cent. curd. This is called 12 per cent. top milk, and by diluting this 5 times, the fats and proteids will be in about the right proportion for an infant up to two weeks. Sugar of milk is added to the extent of 6 per cent. until three months old, then it is made 7 per cent. The quantity of sugar of milk that is already present in the diluted cow's milk can be disregarded in figuring the percentage of milk sugar up to 12 weeks. From birth to 12 weeks, a top milk of 12 per cent. fat and 3 per cent. proteids is used, as the base for modifying the milk. This as before stated, is obtained by letting the milk stand in a cool place, either in a milk bottle or a fruit jar for 5 hours. The upper 1-5 will then represent 12 per cent. fat and 3 per cent. proteids. By siphoning the lower 4-5, the remainder (12 per cent. top milk) is to be used as the base for modification. By diluting this 5 times with boiling water, you will have a 6-10 per cent. proteids and 2 per cent. fat, and then adding 6 per cent. sugar of milk and 5 per cent. lime water, you will have formula No. 1.

Formula No. 2, is made exactly in the same manner, except the top milk is diluted 4 times instead of 5. This formula then represents 8-10 per cent. proteids, 2½ per cent. fats, 5 per cent. alkalinity or lime water, and 6 per cent. sugar of milk. Formula 3 is made by diluting three times. This formula represents 1 per cent. proteids, 3 per cent. fats and 5 per cent. alkalinity, and 6 per cent. sugar of milk. Formula 4 is obtained by using a 9 per cent. top milk, which is obtained by taking the upper ¼ of the bottle of milk, after standing in a cool place for 5 hours. By diluting this 1½ times, there will be produced a 1½ per cent. proteids, 3½ per cent. fats and to this is added 7 per cent. sugar of milk and 5 per cent. lime water. Formula 5 is made by using a 8 per cent. top milk, which is obtained by using the upper 1-3 of the bottle of milk, after standing in a cool place 5 hours, and then diluting with an equal amount of boiling water, and then adding 7 per cent. sugar of milk.

These formulæ provide for the infant to a time when an exclusively milk diet is no longer demanded and to when it can usually take cow's milk undiluted. These formulæ may be modified by the substitution of a thoroughly cooked, thin oatmeal water, in place of the plain water, should the infant be suffering from constipation; or a thoroughly cooked barley water may be substituted for the plain water in case the stools are too loose or frequent.

The paraphernalia required for modifying cow's milk are as many nursing bottles as there are feedings in twenty-four hours, nipples and a quart measure as tall as the nursing bottle for heating the milk, a glass syphon, a 16-ounce graduate, glass or agateware funnel, a bottle brush, non-absorbent cotton, a pitcher for mixing the food, bottle for boracic acid and bicarbonate of soda solutions, and a steriliser. A nursing bottle should be plain, cylindrical, wide-mouthed and graduated to 8 ounces. With these qualities, the bottle is much more easily cleaned and kept sterile. The bottle should be boiled in a solution of soda water and thoroughly rinsed each day. The nipples should be plain and made from pure rubber, with several small openings, large enough to emit the milk, drop by drop, when the bottle is inverted. It is well to have two nipples at hand. The one not in use should be kept in a solution of borax water or boracic acid solution. Once daily, the nipple should be turned inside out and cleaned with a brush and thoroughly rinsed.

In preparing the food, the milk should be dissolved in the required amount of boiling water, using only the best sugar of milk—such as Merck or Squibb—then add the milk, cream and lime water, mixing the whole in a pitcher. A sufficient quantity for 24 hours is always to be prepared at one time. This is then divided into the number of feedings required for one day, each feeding being put in a separate bottle, and each bottle stoppered with cotton. In warm weather, it is quite important that the milk should be pasteurised, *i.e.*, bringing it to the temperature of 167° for one-half hour. It should then be quickly cooled by standing the bottles first in tepid water, then in cold water, and then placed in an ice chest. This rapid cooling is important, as the slow cooling of the milk would give opportunity for the development of the spores which are not destroyed by pasteurising.

The sterilisation of milk is undesirable, as it is rendered more constipating and difficult of digestion, except when it is desired to prepare the milk for several days ahead, and then it is very important that it should be thoroughly sterilised.

With a strict observance of these rules, the mortality of infants deprived of breast milk may be most decidedly diminished, and the infant develop into a strong and healthy child, and better fortified for withstanding the many diseases incident to childhood.

41 OTIS PLACE.

